# Can Chemical Toxicity in Saltwater Be Predicted from Toxicity in Freshwater? A Comprehensive Evaluation Using Species Sensitivity Distributions

**DOI:** 10.1002/etc.5354

**Published:** 2022-05-27

**Authors:** Miina Yanagihara, Kyoshiro Hiki, Yuichi Iwasaki

**Affiliations:** ^1^ Center for Marine Environmental Studies Ehime University Matsuyama Ehime Japan; ^2^ Health and Environmental Risk Research Division National Institute for Environmental Studies Tsukuba Ibaraki Japan; ^3^ Research Institute of Science for Safety and Sustainability National Institute of Advanced Industrial Science and Technology Tsukuba Ibaraki Japan

**Keywords:** Species sensitivity distributions, Marine toxicity tests, Freshwater toxicology, Ecological risk assessment

## Abstract

Species sensitivity distributions (SSDs) play an important role in ecological risk assessment. Estimating SSDs requires toxicity data for many species, but reports on saltwater species are often limited compared to freshwater species. This limitation can constrain informed management of saltwater quality for the protection of marine ecosystems. We investigated the relationships between the parameters (i.e., mean and standard deviation [SD]) of freshwater and saltwater log‐normal SSDs to determine how accurately saltwater toxicity could be estimated from freshwater toxicity test data. We estimated freshwater and saltwater SSDs for 104 chemicals with reported acute toxicity data for five or more species and compared their means, SDs, and hazardous concentrations for 5% of the species (HC5) derived from the acute SSDs. Standard major axis regression analyses generally showed that log–log relationships between freshwater and saltwater SSD means, SDs, and HC5 values were nearly 1:1. In addition, the ratios of freshwater‐to‐saltwater SSD means and HC5 values for most of the 104 chemicals fell within the range 0.1–10. Although such a strong correlation was not observed for SSD SDs (*r* ^2^ < 0.5), differences between freshwater and saltwater SSD SDs were relatively small. These results indicate that saltwater acute SSDs can be reasonably estimated using freshwater acute SSDs. Because the differences of the means and SDs between freshwater and saltwater SSDs were larger when the number of test species used for SSD estimation was lower (i.e., five to seven species in the present study), obtaining toxicity data for an adequate number of species will be key to better approximation of a saltwater acute SSD from a freshwater acute SSD for a given chemical. *Environ Toxicol Chem* 2022;41:2021–2027. © 2022 The Authors. *Environmental Toxicology and Chemistry* published by Wiley Periodicals LLC on behalf of SETAC.

## Graphical abstract

Prediction of saltwater species sensitivity distribution (SSD) from freshwater SSD.

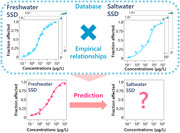

## INTRODUCTION

Environmental impacts of anthropogenic chemicals have been a long‐standing concern worldwide, despite significant progress in their regulation and management (Johnson et al., 2020). A fundamental goal of ecological risk assessment is to estimate the sensitivity of diverse biological species present in multiple habitats such as freshwater and saltwater and thereby enable prediction of the environmental consequences of chemical exposure (De Zwart, 2002; Hiki & Iwasaki, 2020; Maltby et al., 2005; Posthuma et al., 2019; Wheeler et al., 2002b). Species sensitivity distributions (SSDs) have been used to achieve that goal (Posthuma et al., 2002, 2019). Although several different approaches are available to estimate SSDs (Del Signore et al., 2016; Fox et al., 2021; Wheeler et al., 2002a), SSDs are often derived by fitting statistical distributions such as log‐normal distributions to chronic or acute toxicity data (European Food Safety Authority, 2013; Nagai, 2016; Wheeler et al., 2002a). Based on the developed SSD, the concentration that is hazardous for *x*% of the species (HC*x*, typically HC5) is then estimated to derive a predicted‐no‐effect concentration (PNEC); alternatively, the fraction of species adversely affected at a given concentration is estimated to infer the potential ecological risk. For example, the log‐normal SSD has two parameters, the mean and standard deviation (SD); and accurate estimation of not only the mean but also the SD of the distribution is critical for estimating metrics such as HC5 (Sorgog & Kamo, 2019).

Despite many successful applications of SSDs in deriving PNECs and water quality benchmarks (Belanger et al., 2017; Fox et al., 2021), the estimation of SSDs used for regulatory purposes requires toxicity data for relatively large numbers of species. Although the minimum number of required species varies as a function of regulatory jurisdictions (Belanger et al., 2017), several studies have proposed at least 10 species (Carr & Belanger, 2019; Hiki & Iwasaki, 2020; Newman et al., 2000; Wheeler et al., 2002a). This data‐demanding feature is clearly a barrier to the wider application of SSDs because the availability of toxicity data is generally limited for most chemicals (Johnson & Sumpter, 2016; Johnson et al., 2020). Data‐deficient examples include chronic toxicity data (Hoondert et al., 2019) and saltwater toxicity data (Leung et al., 2001; Wheeler et al., 2014a).

Comparisons between freshwater and saltwater toxicity data have been made to address the paucity of saltwater toxicity data (Solbé et al., 1993; De Zwart, 2002; Hutchinson et al., 1998; Klok et al., 2012; Leung et al., 2001; Maltby et al., 2005; Wheeler et al., 2002b, 2014b). These previous studies have generally demonstrated that, on average, the toxicity values obtained from freshwater tests are comparable to those from saltwater tests, and the differences rarely exceed a factor of 10. However, many previous studies have been limited to comparing toxicity values for only a few species in freshwater and saltwater or only the means of the SSDs, despite the importance of the SDs of the SSDs. Furthermore, even when freshwater and saltwater SSDs have been compared, the numbers of chemicals analyzed have often been limited (e.g., 21 chemicals by Wheeler et al. [2002b] and 29 chemicals by Klok et al. [2012]; but see 160 chemicals by De Zwart [2002]).

To provide more thorough evidence for the use of comparisons between freshwater and saltwater toxicity data for ecological risk assessments, we examined the relationships between the mean and SD of log‐normal SSDs estimated from freshwater and saltwater toxicity data for 104 chemicals using a recently developed database (Connors et al., 2019). The log‐normal distribution is one of the most frequently used distributions for estimating SSDs (Wheeler et al., 2002a). The SSD mean and SD are both critical in estimating log‐normal SSDs and thereby the HC5 value; a larger SSD mean indicates a lower toxicity, and a larger SSD SD indicates a greater difference in toxicity values among species (see Supporting Figure S1 for this illustration). Furthermore, we examined whether the differences of those two parameters between freshwater and saltwater SSDs were affected by the modes of action of chemicals and the number of species used for the SSD estimation. These two factors were selected based on the results of previous relevant studies (Del Signore et al., 2016; Hiki & Iwasaki, 2020; Wheeler et al., 2002b).

## MATERIALS AND METHODS

### Ecotoxicity data compilation

An ecotoxicity data set was collected from the EnviroTox database, Ver 1.3.0 (https://envirotoxdatabase.org; Connors et al., 2019) in October 2020. The EnviroTox database has accumulated a wide range of in vivo ecotoxicity data and has been used to estimate SSDs (Hiki & Iwasaki, 2020). The ecotoxicity data obtained from the database included information about the habitats of test organisms (i.e., freshwater or saltwater), and this classification was used to estimate SSDs for freshwater and saltwater species. As described in previous studies (Connors et al., 2019; Kienzler et al., 2019), all chemicals in the EnviroTox database have been classified into three groups based on their modes of action: narcotic, specifically acting, and unclassified. The mode of action classification was based on consensus of four independent models (Verhaar, ASTER, TEST, and OASIS [Kienzler et al., 2019]). The data set obtained from the EnviroTox database included a total of 79,585 test records (68,290 acute and 11,295 chronic) for 1546 species and 3996 chemicals. In the EnviroTox database, the data were curated based on the Stepwise Information‐Filtering Tool method (Beasley et al., 2015) as discussed in Connors et al. (2019), and no additional quality check was performed in the present study.

We adopted the following criteria, which have been modified slightly from Hiki and Iwasaki (2020), to select records to be used for SSD estimation: (1) the effect measures were the median effect concentration or the median lethal concentration (LC50); (2) ecotoxicity data to derive an SSD for a chemical were available for five or more species of both saltwater and freshwater organisms; (3) effect concentrations did not exceed five times the solubility of the tested chemical; (4) the SSDs were based on information about species from at least two of three taxonomic groups (often called *trophic groups* in ecological risk assessment: algae, invertebrates, and fish), and toxicity data for amphibians were not included in our analysis because such data in saltwater were not available in the EnviroTox database; (5) the normality of all SSDs was assessed using the Shapiro‐Wilk test (α = 0.05 with Holm's *p*‐value adjustment), and based on that test, the assumption of normality was not rejected. The chronic records were not used for the analysis in the present study because the number of records was limited after the selection and only six SSDs were estimated. Based on these criteria, 5838 out of 68,290 acute test records (4378 and 1460 for freshwater and saltwater species, respectively) were selected. According to the fifth criterion (i.e., the Shapiro‐Wilk test), 13 chemicals were excluded from our analysis. The records contained LC50 values of 854 freshwater species and 296 saltwater species for 104 chemicals.

### Data analysis

We conducted data analysis using R, Ver 3.6.3 (R Core Team, 2020), and the R package “tidyverse” (Wickham et al., 2019); the data visualization was performed with the R package “ggplot2” (Wickham, 2016).

The SSD estimation was performed as described in Hiki and Iwasaki (2020) with slight modifications. If more than one effect concentration was available for a given combination of species, chemicals, and habitats, their geometric means were calculated and used. The mean and SD of the log‐normal SSDs were estimated from the log_10_‐transformed effect concentrations using the “mean” and “sd” functions in the R packages “base” and “stats,” respectively. Log_10_‐transformed values of acute HC5 for individual chemicals were estimated using the following equation (Aldenberg et al., 2001)

(1) HC5=mean−1.645×SD

In Equation 1, mean and SD are the log_10_‐transformed mean and SD of the SSD. Because only acute toxicity data were used in our analysis, hereafter, acute HC5 is referred to as HC5 for simplicity.

The relationships of the means, SDs, and HC5 values between freshwater and saltwater SSDs were analyzed by standardized major axis (SMA) regression using the R package “smatr” (Warton et al., 2012). Because a 10‐fold difference of means or a 4‐fold difference of SDs between freshwater and saltwater SSDs (i.e., differences of no more than ±1 or ±0.6 on a log_10_ scale, respectively) lead to an approximately 10‐fold difference of HC5 values (Equation 1), differences this large were operationally considered as thresholds for comparing means and SDs in the present study. We conducted a complementary analysis to determine whether metals such as cadmium and copper, the toxicities of which are known to vary with salinity (Hall & Anderson, 1995), showed characteristic relationships between freshwater and saltwater SSDs. The results based on SSDs estimated for 11 metals were similar to those based on 104 chemicals (see Supporting Figure S2A–C), although the influences of other important water quality parameters such as pH, dissolved organic matter, and other cations (Adams et al., 2020; Arnold et al., 2005) were not taken into account in this analysis. Thus, metals were not analyzed independently from the 104 chemicals in the main text.

The influence of the number of species on differences in the two parameters (i.e., mean and SD) between freshwater and saltwater SSDs was examined by calculating Pearson's correlation coefficient. The lower of the two numbers of the tested species for a specific chemical in freshwater and saltwater SSDs was used to assess the influence of the number of species. Hereafter, we refer to the lower of the two numbers of the species as the number of species, unless otherwise stated. The influence of the modes of action was also investigated by using a one‐sample *t* test to determine whether the mean ratios of freshwater‐to‐saltwater SSD parameters deviated from 1 for each mode of action.

## RESULTS AND DISCUSSION

### Overview of estimated SSDs

Table 1 summarizes the information on the 104 selected chemicals and estimated SSDs. A list of 104 chemicals and the estimated SSD parameters is provided in Supporting Table S1. The number of chemicals for which we could estimate SSDs was more than three times larger than the numbers of chemicals examined in two previous studies (≤29 [Klok et al., 2012; Wheeler et al., 2002b]). Although the number of chemicals investigated by De Zwart (2002), who evaluated the relationship between freshwater and saltwater SSD means based on acute toxicity data, was larger than ours, De Zwart generally adopted less strict criteria for estimating SSDs (e.g., four or more species). Of the 104 chemicals, 20, 39, and 45 were assigned to the narcotic, specifically acting, and unclassified groups, respectively. The average number of species in freshwater SSDs was approximately three times the number in saltwater SSDs, and in both cases the number of species per SSD varied by more than an order of magnitude (see Table 1). Two and three taxonomic groups were included in estimating 38% and 62% of freshwater SSDs, respectively. Fifty‐one percent and 49% of saltwater SSDs included two and three taxonomic groups, respectively. Supporting Table S2 provides detailed information about the taxonomic composition of the SSDs for the different mode of action groups.

**Table 1:** Summary information about the number of chemicals examined and the number of species in the species sensitivity distributions used in the present study^a^

		Mode of action		
	Total	Narcotic	Specifically acting	Unclassified
Number of chemicals	104	20	39	45
Number of species per freshwater SSD	42 ± 45 (5–256)	30 ± 38 (5–183)	41 ± 34 (8–174)	49 ± 56 (7–256)
Number of species per saltwater SSD	14 ± 19 (5–120)	8 ± 6 (5–28)	10 ± 6 (5–26)	20 ± 27 (5–120)

^a^ Mean ± standard deviation; minimum–maximum.

SSD = species sensitivity distribution.

### Comparison of SSD means, SDs, and HC5 values

**Figure 1:**
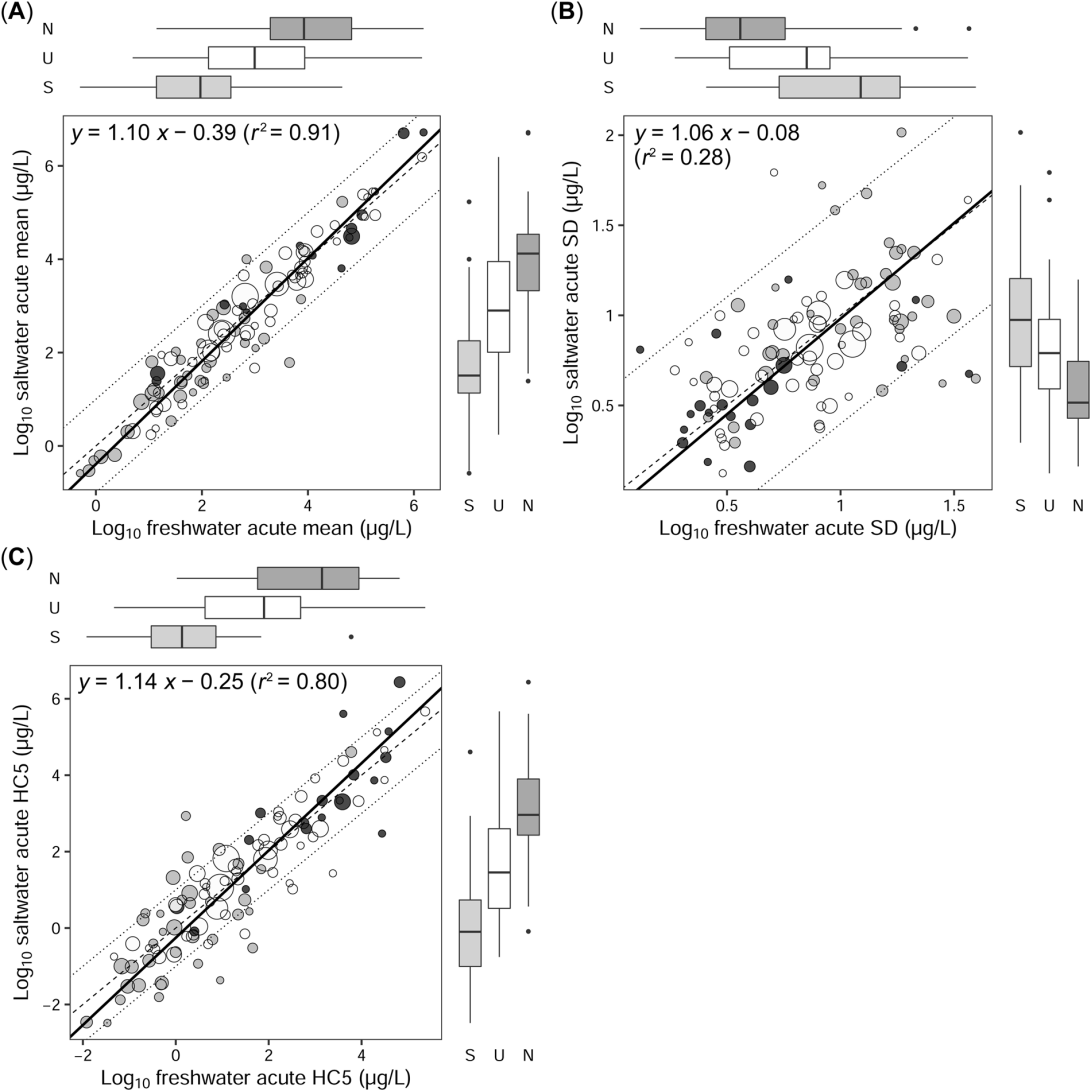
Relationships between the (**A**) means, (**B**) standard deviations, and (**C**) hazardous concentrations for 5% of the species of freshwater and saltwater species sensitivity distributions (SSDs). White, gray, and black dots (and box plots) represent unclassified, specifically acting, and narcotic chemicals, respectively. (**A**,**C**) The dashed line indicates a 1:1 (freshwater/saltwater) ratio, and the dotted lines indicate ratios of 1:10 and 10:1 in actual values. (**B**) The dashed line indicates a 1:1 (freshwater/saltwater) ratio, and the dotted lines indicate ratios of 4:1 and 1:4 in actual values. The bold line in each panel shows the estimated standard major axis regression model. Dot sizes show the number of test species in the freshwater or saltwater SSDs, whichever is lower. Box plots show the distributions of freshwater (top) and saltwater (right) SSD parameters for each of the three modes of action. The bold horizontal line, box, error bar, and black circle indicate the median, interquartile range, 1.5× (interquartile range), and outlier, respectively. N = narcotic; U = unclassified group; S = specifically acting; SD = standard deviation; HC5 = hazardous concentration for 5% of the species.

The slope of the SMA regression of log_10_‐transformed freshwater versus saltwater SSD means was 1.10 (95% confidence interval [CI] 1.04–1.17), and the intercept was −0.39 (95% CI −0.60 to −0.18; *r* ^2^ = 0.91; Figure 1A). If only SSDs with at least 10 test species (Hiki & Iwasaki, 2020) were analyzed, a similar slope (1.10, 95% CI 1.00–1.21) and intercept (−0.23, 95% CI −0.49 to 0.04) were obtained (Supporting Figure S3A); but their 95% CIs included 1 and 0, respectively. These results suggest that the freshwater and saltwater SSD means were not clearly different. In addition, the ratios of freshwater‐to‐saltwater SSD means fell within the range 0.1–10 for 96% of the chemicals examined, except for four chemicals (trichlorfon, methidathion, 1,2,3,4,5,6‐hexachlorocyclohexane, and ethoprop) for which there were fewer than 10 test species. These results suggest that the mean of a freshwater SSD for a chemical can be used as an approximation of the mean of the saltwater SSD, although the two may differ by as much as a factor of 10 (see below for more detailed discussion).

Likewise, the fact that the slope of the SMA regression of log_10_‐transformed SSD SDs was 1.06 (95% CI 0.90–1.25) and the intercept was −0.08 (95% CI −0.25 to 0.09) suggested that the freshwater and saltwater SSD SDs were not clearly different (Figure 1B). Similar results were obtained if the analysis was based on SSDs with ≥10 tested species (Supporting Figure S3B). Even though the *r* ^2^ values were relatively low in these analyses (<0.5; Figures 1B; Supporting Figure S3B), the ratios of freshwater‐to‐saltwater SSD SDs for 92% and 100% of the chemicals we examined fell within the range 0.25–4 when the numbers of tested species were ≥5 and ≥10, respectively. This is consistent with previous findings that the variation in SSD SDs decreased with increasing numbers of species (De Zwart, 2002; Hendriks et al., 2013; Hiki & Iwasaki, 2020). Although similar relationships have been obtained for acute‐to‐chronic SSD SDs (Hiki & Iwasaki, 2020), our results provide the first evidence that the SD of the freshwater acute SSD for a chemical can be used as an approximation of the SD of the saltwater acute SSD.

The slope and intercept of the SMA regression of log_10_‐transformed HC5 values were 1.14 (95% CI 1.05–1.25) and −0.25 (95% CI −0.48 to −0.03), respectively (*r* ^2^ = 0.80; Figure 1C). If only SSDs with ≥10 test species were analyzed (Supporting Figure S3C), the estimated slope and intercept were 1.13 (95% CI 0.97–1.30) and 0.004 (95% CI −0.25 to 0.26), respectively. The implication was that there was no clear difference between the freshwater and saltwater HC5 values. The ratios of saltwater‐to‐freshwater HC5 values for 23 chemicals (22% of the 104 chemicals examined) fell outside the range 0.1–10. Saltwater HC5 values were smaller than freshwater HC5 values for 15 out of the 23 chemicals (see Supporting Table S3 for more detailed information). For example, HC5 values for 1,2,3,4,5,6‐hexachlorocyclohexane in freshwater and saltwater SSDs were estimated to be 9.0 and 0.043 µg/L, respectively, resulting in the smallest HC5 value ratio of 0.0048. Fourteen of the 15 ratios that were <0.1 (i.e., cases for which a saltwater HC5 value would be overestimated from a freshwater HC5 value) were based on saltwater SSDs with eight or fewer test species. Together with similar findings from previous studies (Klok et al., 2012; Wheeler et al., 2002b), the results from the present study suggest that the HC5 value estimated from a freshwater acute SSD can be used as a first approximation of the saltwater acute HC5 value. If a conservative estimate of a saltwater acute HC5 value for a chemical is required, we recommend multiplication of the freshwater acute HC5 value by 0.1, particularly when the freshwater SSD includes eight or more test species, as discussed in the next section.

### Influence of the number of species and modes of action of chemicals

**Figure 2:**
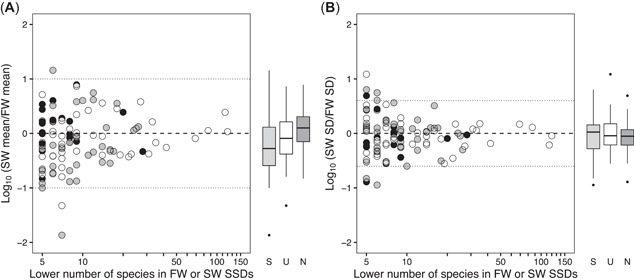
Relationships between the lower number of species in the freshwater or saltwater species sensitivity distributions (SSDs) and the ratios of saltwater‐to‐freshwater SSD (**A**) means and (**B**) standard deviations (SDs). White, gray, and black dots (and box plots) represent unclassified, specifically acting, and narcotic chemicals, respectively. Dashed lines indicate ratios of 1 in both panels, and dotted lines indicate ratios of 10 and 0.1 for means and of 0.25 and 4 for SDs. Box plots show the distributions of the ratios for the three modes of action. The bold horizontal line, box, error bar, and black circle indicate the median, interquartile range, 1.5× (interquartile range), and outlier, respectively. SW = saltwater; FW = freshwater; S = specifically acting; U = unclassified group; N = narcotic.

As the number of species used for estimating SSDs increased, the ratios of the saltwater‐to‐freshwater SSD means and SDs approached 1.0 (Figure 2). Indeed, there were weak but statistically significant correlations between the number of species and the absolute values of the ratios of the saltwater‐to‐freshwater SSD means and SDs (Pearson's *r* = −0.20 and −0.22, respectively, *p* < 0.05; Figure 2). Also, for the chemicals with SSDs estimated from eight or more test species, the saltwater‐to‐freshwater ratios of SSD means were in the range 0.1–10, and those of SSD SDs were in the range of 0.25–4 (Figure 2). These results highlight the importance of using results from more than a few test species to estimate the parameters of an SSD and thereby reduce the uncertainty in predicting a saltwater SSD from a freshwater SSD.

The saltwater‐to‐freshwater ratios of the two parameters (mean and SD) of the SSDs varied considerably for all three modes of actions (see the boxplots in Figure 2). Except for the saltwater‐to‐freshwater ratios of the SSD mean for specifically acting chemicals, there was no statistically significant deviation of the ratios from 1 (i.e., from 0 of the log_10_‐transformed ratios). For the specifically acting chemicals, the mean of the logarithms of the ratios of the saltwater‐to‐freshwater SSD means was significantly less than 0. The implication was that, on average, the means of the saltwater SSDs were lower than those of the freshwater SSDs. However, this significant difference was not apparent when SSDs with ≥10 tested species were analyzed. The implication was that the significant deviation detected in the ratios for the specifically acting chemicals may have been an artifact of the small number of species. Among the four chemicals with ratios of saltwater‐to‐freshwater SSD means that differed by more than a factor of 10 (Supporting Figure S4A), three were insecticides and categorized into the specifically acting mode of action group (i.e., trichlorfon, methidathion, and 1,2,3,4,5,6‐hexachlorocyclohexane). The biased proportion of the sensitive taxonomic group (i.e., arthropods) in freshwater and saltwater SSDs of the three chemicals (Supporting Figure S4) might have resulted in the large difference in SSD means. Similarly, five of eight chemicals with SD ratios outside the range 0.25–4 (Supporting Figure S4B) were specifically acting chemicals (i.e., trichlorfon, 1,2,3,4,5,6‐hexachlorocyclohexane, ethion, chlorpyrifos‐methyl, and fonofos). Although further examination is required, these results suggest that specifically acting chemicals may exhibit larger differences between freshwater and saltwater SSD parameters, particularly when the number of test species is low.

### Implications and future research needs

We investigated relationships between freshwater and saltwater acute SSDs for 104 chemicals by focusing on the means and SDs of their log‐normal SSDs. The log–log relationships between the freshwater and saltwater SSD means, SDs, and HC5 values were generally close to the 1:1 line. In addition, the means and HC5 values of the freshwater and saltwater species generally differed by less than a factor of 10, and the corresponding SDs differed by less than a factor of 4 for the majority of chemicals that we examined. These results indicate that a saltwater SSD for a chemical can be reasonably estimated from the corresponding freshwater SSD when there are few or no relevant ecotoxicity data for saltwater. Despite previous studies that have reported changes in bioavailability and toxicity with salinity (Borecka et al., 2016; Dutton & Fisher, 2011; Nero et al., 2006), our results, together with those from similar previous studies (De Zwart, 2002; Klok et al., 2012; Wheeler et al., 2002b), imply that systematic differences are undetectable when comparisons are made of the sensitivity of multiple species based on SSDs. The explanation may be that the magnitude and direction of the changes of toxicity with salinity (e.g., toxicity increase or decrease) vary among species and chemicals (Hall & Anderson, 1995; van Wezel, 1998). However, the variations observed when comparing parameters derived from freshwater and saltwater SSDs (e.g., a factor of 10 for SSD means) may be at least partly explained by changes of toxicity with salinity.

In addition, relationships between freshwater and saltwater SSDs estimated based on chronic toxicity data were not investigated in the present study because of the limited chronic toxicity data, particularly in saltwater. Future work should examine such relationships for chronic SSDs to further test the applicability of the extrapolation of freshwater SSDs to saltwater SSDs. Moreover, because statistical distributions other than the log‐normal distribution are often adopted in estimating SSDs, the applicability of our findings in such cases should be tested. More thorough data collection will be required to better capture the relationships between freshwater and saltwater SSDs and to best take advantage of freshwater toxicity data in saltwater ecological risk assessments.

## Supporting Information

The Supporting Information is available on the Wiley Online Library at https://doi.org/10.1002/etc.5354.

## Disclaimer

All authors declare no conflict of interest.

## Author Contributions Statement

**Miina Yanagihara**: Conceptualization; Formal analysis; Funding acquisition; Investigation; Methodology; Software; Writing—original draft; Writing—review & editing. **Kyoshiro Hiki**: Conceptualization; Investigation; Methodology; Software; Writing—review & editing. **Yuichi Iwasaki**: Conceptualization; Funding acquisition; Investigation; Methodology; Writing—original draft; Writing—review & editing.

## Supporting information

This article includes online‐only Supporting Information.

Supporting information.

Supporting information.

## Data Availability

The data used in the present study can be obtained from the EnviroTox database (http://www.envirotoxdatabase.org/). A list of species sensitivity distribution parameters estimated for 104 chemicals is available in Supporting Data, Table S1. Data, associated metadata, and calculation tools are also available from the corresponding author (yuichiwsk@gmail.com).

## References

[etc5354-bib-0001] Adams, W. , Blust, R. , Dwyer, R. , Mount, D. , Nordheim, E. , Rodriguez, P. H. , & Spry, D. (2020). Bioavailability assessment of metals in freshwater environments: A historical review. Environmental Toxicology and Chemistry, 39(1), 48–59. 31880839 10.1002/etc.4558 PMC11382335

[etc5354-bib-0002] Aldenberg, T. , Jaworska, J. S. , & Traas, T. P. (2001). Normal species sensitivity distributions and probabilistic ecological risk assessment. In L. Posthuma , G. W. Suter II , & T. P. Traas (Eds.), Species sensitivity distributions in ecotoxicology (pp. 49–102). CRC Press.

[etc5354-bib-0003] Arnold, W. R. , Santore, R. C. , & Cotsifas, J. S. (2005). Predicting copper toxicity in estuarine and marine waters using the biotic ligand model. Marine Pollution Bulletin, 50(12), 1634–1640. 16040053 10.1016/j.marpolbul.2005.06.035

[etc5354-bib-0004] Beasley, A. , Belanger, S. E. , & Otter, R. R. (2015). Stepwise Information‐Filtering Tool (SIFT): A method for using risk assessment metadata in a nontraditional way. Environmental Toxicology and Chemistry, 34(6), 1436–1442. 25728797 10.1002/etc.2955

[etc5354-bib-0005] Belanger, S. , Barron, M. , Craig, P. , Dyer, S. , Galay‐Burgos, M. , Hamer, M. , Marshall, S. , Posthuma, L. , Raimondo, S. , & Whitehouse, P. (2017). Future needs and recommendations in the development of species sensitivity distributions: Estimating toxicity thresholds for aquatic ecological communities and assessing impacts of chemical exposures. Integrated Environmental Assessment and Management, 13(4), 664–674. 27531323 10.1002/ieam.1841 PMC6116543

[etc5354-bib-0006] Borecka, M. , Białk‐Bielińska, A. , Haliński, Ł. P. , Pazdro, K. , Stepnowski, P. , & Stolte, S. (2016). The influence of salinity on the toxicity of selected sulfonamides and trimethoprim towards the green algae *Chlorella vulgaris* . Journal of Hazardous Materials, 308, 179–186. 26835894 10.1016/j.jhazmat.2016.01.041

[etc5354-bib-0007] Carr, G. J. , & Belanger, S. E. (2019). SSDs revisited: Part I—A framework for sample size guidance on species sensitivity distribution analysis. Environmental Toxicology and Chemistry, 38(7), 1514–1525. 30994946 10.1002/etc.4445

[etc5354-bib-0008] Connors, K. A. , Beasley, A. , Barron, M. G. , Belanger, S. E. , Bonnell, M. , Brill, J. L. , de Zwart, D. , Kienzler, A. , Krailler, J. , Otter, R. , Phillips, J. L. , & Embry, M. R. (2019). Creation of a curated aquatic toxicology database: EnviroTox. Environmental Toxicology and Chemistry, 38(5), 1062–1073. 30714190 10.1002/etc.4382 PMC6850623

[etc5354-bib-0009] Del Signore, A. , Hendriks, A. J. , Lenders, H. J. R. , Leuven, R. S. E. W. , & Breure, A. M. (2016). Development and application of the SSD approach in scientific case studies for ecological risk assessment. Environmental Toxicology and Chemistry, 35(9), 2149–2161. 27144499 10.1002/etc.3474

[etc5354-bib-0010] De Zwart, D. (2002). Observed regularities in species sensitivity distributions for aquatic species. In L. Posthuma , G. W. Suter II, & T. P. Traas (Eds.), *Species sensitivity distributions in ecotoxicology* (pp. 133–154). CRC Press.

[etc5354-bib-0011] Dutton, J. , & Fisher, N. S. (2011). Salinity effects on the bioavailability of aqueous metals for the estuarine killifish *Fundulus heteroclitus* . Environmental Toxicology and Chemistry, 30(9), 2107–2114. 21688308 10.1002/etc.600

[etc5354-bib-0012] European Food Safety Authority . (2013). Guidance on tiered risk assessment for plant protection products for aquatic organisms in edge‐of‐field surface waters. EFSA Journal, 11(7), Article 3290.

[etc5354-bib-0013] Fox, D. R. , van Dam, R. A. , Fisher, R. , Batley, G. E. , Tillmanns, A. R. , Thorley, J. , Schwarz, C. J. , Spry, D. J. , & McTavish, K. (2021). Recent developments in species sensitivity distribution modeling. Environmental Toxicology and Chemistry, 40(2), 293–308. 33170526 10.1002/etc.4925

[etc5354-bib-0014] Hall, L. W. , & Anderson, R. D. (1995). The influence of salinity on the toxicity of various classes of chemicals to aquatic biota. *Critical Reviews in Toxicology*, 25(4), 281–346. 10.3109/10408449509021613 7576155

[etc5354-bib-0015] Hendriks, A. J. , Awkerman, J. A. , de Zwart, D. , & Huijbregts, M. A. J. (2013). Sensitivity of species to chemicals: Dose–response characteristics for various test types (LC50, LR50 and LD50) and modes of action. Ecotoxicology and Environmental Safety, 97, 10–16. 23932508 10.1016/j.ecoenv.2013.06.020

[etc5354-bib-0016] Hiki, K. , & Iwasaki, Y. (2020). Can we reasonably predict chronic species sensitivity distributions from acute species sensitivity distributions? Environmental Science & Technology, 54(20), 13131–13136. 32924457 10.1021/acs.est.0c03108

[etc5354-bib-0017] Hoondert, R. P. J. , Oldenkamp, R. , de Zwart, D. , van de Meent, D. , & Posthuma, L. (2019). QSAR‐based estimation of species sensitivity distribution parameters: An exploratory investigation. Environmental Toxicology and Chemistry, 38(12), 2764–2770. 31553801 10.1002/etc.4601 PMC6900027

[etc5354-bib-0018] Hutchinson, T. H. , Scholz, N. , & Guhl, W. (1998). Analysis of the ECETOC Aquatic Toxicity (EAT) database. IV—Comparative toxicity of chemical substances to freshwater versus saltwater organisms. Chemosphere, 36(1), 143–153.

[etc5354-bib-0019] Johnson, A. C. , Jin, X. , Nakada, N. , & Sumpter, J. P. (2020). Learning from the past and considering the future of chemicals in the environment. Science, 367(6476), 384–387. 31974243 10.1126/science.aay6637

[etc5354-bib-0020] Johnson, A. C. , & Sumpter, J. P. (2016). Are we going about chemical risk assessment for the aquatic environment the wrong way? Environmental Toxicology and Chemistry, 35(7), 1609–1616. 27331653 10.1002/etc.3441

[etc5354-bib-0021] Kienzler, A. , Connors, K. A. , Bonnell, M. , Barron, M. G. , Beasley, A. , Inglis, C. G. , Norberg‐King, T. J. , Martin, T. , Sanderson, H. , Vallotton, N. , Wilson, P. , & Embry, M. R. (2019). Mode of action classifications in the EnviroTox database: Development and implementation of a consensus MOA classification. Environmental Toxicology and Chemistry, 38(10), 2294–2304. 31269286 10.1002/etc.4531 PMC6851772

[etc5354-bib-0022] Klok, C. , de Vries, P. , Jongbloed, R. , & Tamis, J. (2012). Literature review on the sensitivity and exposure of marine and estuarine organisms to pesticides in comparison to corresponding fresh water species. EFSA Supporting Publications, 9(11), Article 357. 10.2903/sp.efsa.2012.en-357

[etc5354-bib-0023] Leung, K. M. Y. , Morritt, D. , Wheeler, J. R. , Whitehouse, P. , Sorokin, N. , Toy, R. , Holt, M. , & Crane, M. (2001). Can saltwater toxicity be predicted from freshwater data? Marine Pollution Bulletin, 42(11), 1007–1013. 11763210 10.1016/s0025-326x(01)00135-7

[etc5354-bib-0024] Maltby, L. , Blake, N. , Brock, T. C. M. , & Van Den Brink, P. J. (2005). Insecticide species sensitivity distributions: Importance of test species selection and relevance to aquatic ecosystems. Environmental Toxicology and Chemistry, 24(2), 379–388. 15719998 10.1897/04-025r.1

[etc5354-bib-0025] Nagai, T. (2016). Ecological effect assessment by species sensitivity distribution for 68 pesticides used in Japanese paddy fields. Journal of Pesticide Science, 41(1), 6–14. 30364873 10.1584/jpestics.D15-056 PMC6200050

[etc5354-bib-0026] Nero, V. , Farwell, A. , Lee, L. E. J. , Van Meer, T. , MacKinnon, M. D. , & Dixon, D. G. (2006). The effects of salinity on naphthenic acid toxicity to yellow perch: Gill and liver histopathology. Ecotoxicology and Environmental Safety, 65(2), 252–264. 16129489 10.1016/j.ecoenv.2005.07.009

[etc5354-bib-0027] Newman, M. C. , Ownby, D. R. , Mézin, L. C. A. , Powell, D. C. , Christensen, T. R. L. , Lerberg, S. B. , & Anderson, B. A. (2000). Applying species‐sensitivity distributions in ecological risk assessment: Assumptions of distribution type and sufficient numbers of species. Environmental Toxicology and Chemistry, 19(2), 508–515.

[etc5354-bib-0028] Posthuma, L , Suter, G. W., II , & Traas, T. P. (2002). Species sensitivity distributions in ecotoxicology. CRC Press.

[etc5354-bib-0029] Posthuma, L. , van Gils, J. , Zijp, M. C. , van de Meent, D. , & de Zwart, D. (2019). Species sensitivity distributions for use in environmental protection, assessment, and management of aquatic ecosystems for 12 386 chemicals. Environmental Toxicology and Chemistry, 38(4), 905–917. 10.1002/etc.4373 30675920 PMC6907411

[etc5354-bib-0030] R Core Team . (2020). *R: A language and environment for statistical computing*. https://www.R-project.org/

[etc5354-bib-0031] Solbé, J. F. de L. G. , Buyle, B. , Guhl, W. , Hutchinson, T. , Laenge, R. , Mark, U. , Munk, R. , & Scholz, N. (1993). Developing hazard identification for the aquatic environment. Science of the Total Environment, 134, 47–61.

[etc5354-bib-0032] Sorgog, K. , & Kamo, M. (2019). Quantifying the precision of ecological risk: Conventional assessment factor method vs. species sensitivity distribution method. Ecotoxicology and Environmental Safety, 183, Article 109494. 31376805 10.1016/j.ecoenv.2019.109494

[etc5354-bib-0033] van Wezel, A. P. (1998). Chemical and biological aspects of ecotoxicological risk assessment of ionizable and neutral organic compounds in fresh and marine waters: A review. Environmental Reviews, 6(2), 123–137.

[etc5354-bib-0034] Warton, D. I. , Duursma, R. A. , Falster, D. S. , & Taskinen, S. (2012). smatr 3—An R package for estimation and inference about allometric lines. Methods in Ecology and Evolution, 3, 257–259.

[etc5354-bib-0035] Wheeler, J. R. , Grist, E. P. M. , Leung, K. M. Y. , Morritt, D. , & Crane, M. (2002a). Species sensitivity distributions: Data and model choice. Marine Pollution Bulletin, 45(1–12), 192–202. 12398385 10.1016/s0025-326x(01)00327-7

[etc5354-bib-0036] Wheeler, J. R. , Leung, K. M. Y. , Morritt, D. , Sorokin, N. , Rogers, H. , Toy, R. , Holt, M. , Whitehouse, P. , & Crane, M. (2002b). Freshwater to saltwater toxicity extrapolation using species sensitivity distributions. Environmental Toxicology and Chemistry, 21(11), 2459–2467. 12389927

[etc5354-bib-0037] Wheeler, J. R. , Maynard, S. K. , & Crane, M. (2014a). Are acute and chronic saltwater fish studies required for plant protection and biocidal product active substance risk assessment? Environmental Toxicology and Chemistry, 33(3), 703–707. 10.1002/etc.2478 24288251

[etc5354-bib-0038] Wheeler, J. R. , Maynard, S. K. , & Crane, M. (2014b). An evaluation of fish early life stage tests for predicting reproductive and longer‐term toxicity from plant protection product active substances. Environmental Toxicology and Chemistry, 33(8), 1874–1878. 24799351 10.1002/etc.2630

[etc5354-bib-0039] Wickham, H. (2016). ggplot2: Elegant graphics for data analysis. Springer‐Verlag.

[etc5354-bib-0040] Wickham, H. , Averick, M. , Bryan, J. , Chang, W. , McGowan, L. , François, R. , Grolemund, G. , Hayes, A. , Henry, L. , Hester, J. , Kuhn, M. , Pedersen, T. , Miller, E. , Bache, S. , Müller, K. , Ooms, J. , Robinson, D. , Seidel, D. , Spinu, V. , … Yutani, H. (2019). Welcome to the {tidyverse}. Journal of Open Source Software, 4(43), Article 1686.

