# Supplementary material for: Can Chemical Toxicity in Saltwater Be Predicted from Toxicity in Freshwater? A Comprehensive Evaluation Using Species Sensitivity Distributions
Source: Environ Toxicol Chem. 2022 May 27;41(8):2021–7. doi: 10.1002/etc.5354 (PMC9542858; doi:10.1002/etc.5354)
Supplement: Supplementary file 1 — Supporting information. [file ETC-41-2021-s002.pdf]

Supporting Information for “Can chemical toxicity in saltwater be predicted from toxicity in freshwater? A comprehensive evaluation using species sensitivity distributions”

Authors: Miina Yanagihara,[a] Kyoshiro Hiki,[b] and Yuichi Iwasaki[c,]*

[a]Center for Marine Environmental Studies, Ehime University, Matsuyama, Ehime 790-8577, Japan
[b]Health and Environmental Risk Research Division, National Institute for Environmental Studies, Tsukuba, Ibaraki 305-8506, Japan
[c]Research Institute of Science for Safety and Sustainability, National Institute of Advanced Industrial Science and Technology, Tsukuba, Ibaraki 305-8569, Japan

* Address correspondence to yuichiwsk@gmail.com

List of materials

Table S2
Table S3
Figure S1
Figure S2
Figure S3
Figure S4

TABLE S2: Median proportions (%) of each taxonomic group in freshwater and saltwater species sensitivity distributions (SSDs) for chemicals with three different modes of action (minimum–maximum)

		Mode of action		
		Narcotic	Specifically acting	Unclassified
Freshwater	Algae	14 (0–50)	0 (0–32)	9 (0–72)
	Fish	44 (25–76)	53 (17–82)	46 (11–86)
	Invertebrate	36 (17–72)	43 (13–83)	40 (5–64)
Saltwater	Algae	17 (0–60)	0 (0–33)	9 (0–80)
	Fish	22 (13–60)	52 (20–88)	33 (14–83)
	Invertebrate	60 (20–83)	44 (0–80)	56 (0–84)

TABLE S3: List of chemicals for which the ratios of freshwater-to-saltwater SSD HC5s were not within the 10-fold ranges.

Chemical	SSD mean (log_{10}-transformed) [µg/L]		SSD SD (log_{10}-transformed) [µg/L]		SSD HC5 (log_{10}-transformed) [µg/L]		HC5 ratio (SW/FW)	Number of tested species		MoA
	FW	SW	FW	SW	FW	SW		FW	SW	
1,2,3,4,5,6-Hexachlorocyclohexane	2.47	1.47	0.92	1.72	0.96	-1.37	0.0048	23	5	S
Methidathion	3.65	1.79	1.21	1.40	1.66	-0.52	0.0066	25	7	S
Bromoform	4.63	3.80	0.12	0.81	4.44	2.47	0.011	5	5	N
MCPA	4.55	4.38	0.71	1.79	3.38	1.43	0.011	18	5	U
Ethoprop	2.99	1.67	0.92	1.11	1.49	-0.15	0.023	7	7	U
Dibutyl phthalate	3.29	2.66	0.47	1.00	2.52	1.02	0.032	17	7	U
Chlorpyrifos-methyl	1.72	1.51	1.27	2.02	-0.36	-1.81	0.036	19	6	S
Fonofos	2.08	1.67	0.98	1.58	0.48	-0.93	0.039	8	6	S
Benzyl butyl phthalate	3.01	2.57	0.33	0.85	2.45	1.17	0.053	9	5	U
Phorate	1.42	0.53	1.05	1.23	-0.31	-1.48	0.068	19	8	S
Thiodicarb	3.01	2.10	0.87	1.01	1.58	0.44	0.071	8	5	S
Permethrin	0.60	0.30	0.55	1.06	-0.30	-1.44	0.074	68	16	S
Technical chlordane	1.14	0.72	0.27	0.69	0.70	-0.42	0.077	25	6	U
Phosmet	2.63	2.46	1.11	1.68	0.79	-0.30	0.081	21	8	S
Deltamethrin	-0.30	-0.58	0.71	1.15	-1.47	-2.48	0.098	31	5	S
2-Butenoic acid, 3-[(dimethoxyphosphinyl)oxy]-, methyl ester	1.51	2.02	1.32	0.99	-0.66	0.39	10.98	17	6	S
Dimethoate	3.21	3.83	1.38	1.08	0.93	2.06	13.29	65	12	S

Diquat dibromide	3.91	4.19	1.27	0.72	1.82	3.01	15.39	36	7	N
Dichlorvos	2.41	2.96	1.5	1	-0.06	1.32	23.97	64	18	S
Phosphamidon	2.2	2.81	1.18	0.58	0.26	1.85	39.11	17	10	S
Glycine, N,N-bis(carboxymethyl)-, trisodium salt	5.81	6.7	0.6	0.16	4.82	6.43	41.27	13	9	N
Methanol	6.18	6.72	1.57	0.68	3.61	5.61	99.73	20	5	N
Trichlorfon	2.84	4	1.6	0.65	0.22	2.93	518.6	72	6	S

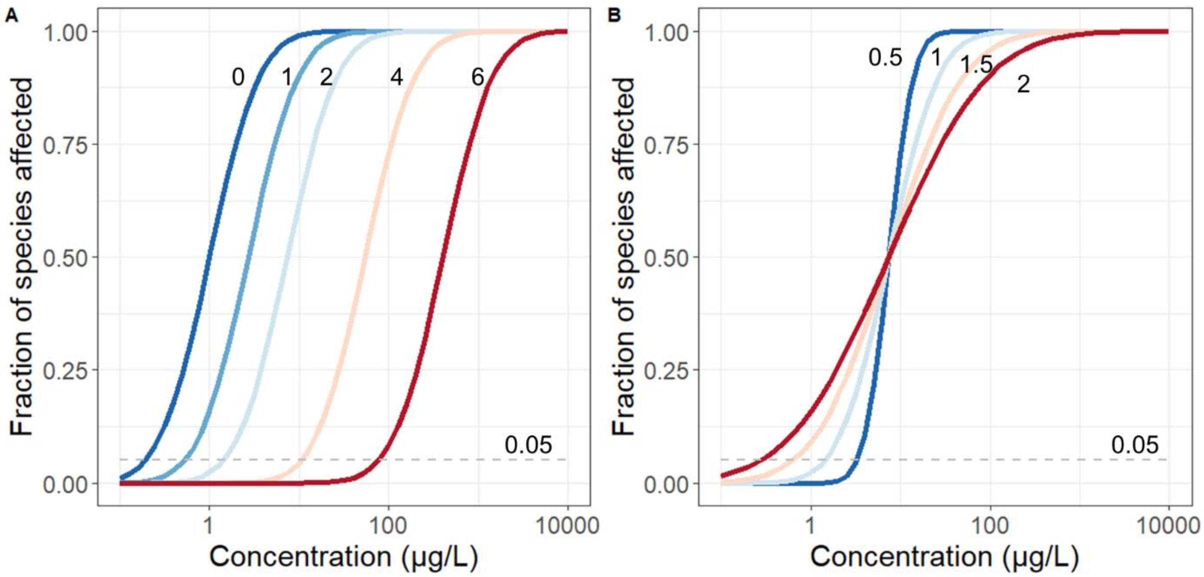

FIGURE S1: Cumulative lognormal distribution to illustrate the effects of the mean (A) and SD (B). (A) The distribution with various log_{10}Mean values and a log_{10}SD of 1; (B) The distribution with a log_{10}Mean of 2 and various log_{10}SD values.

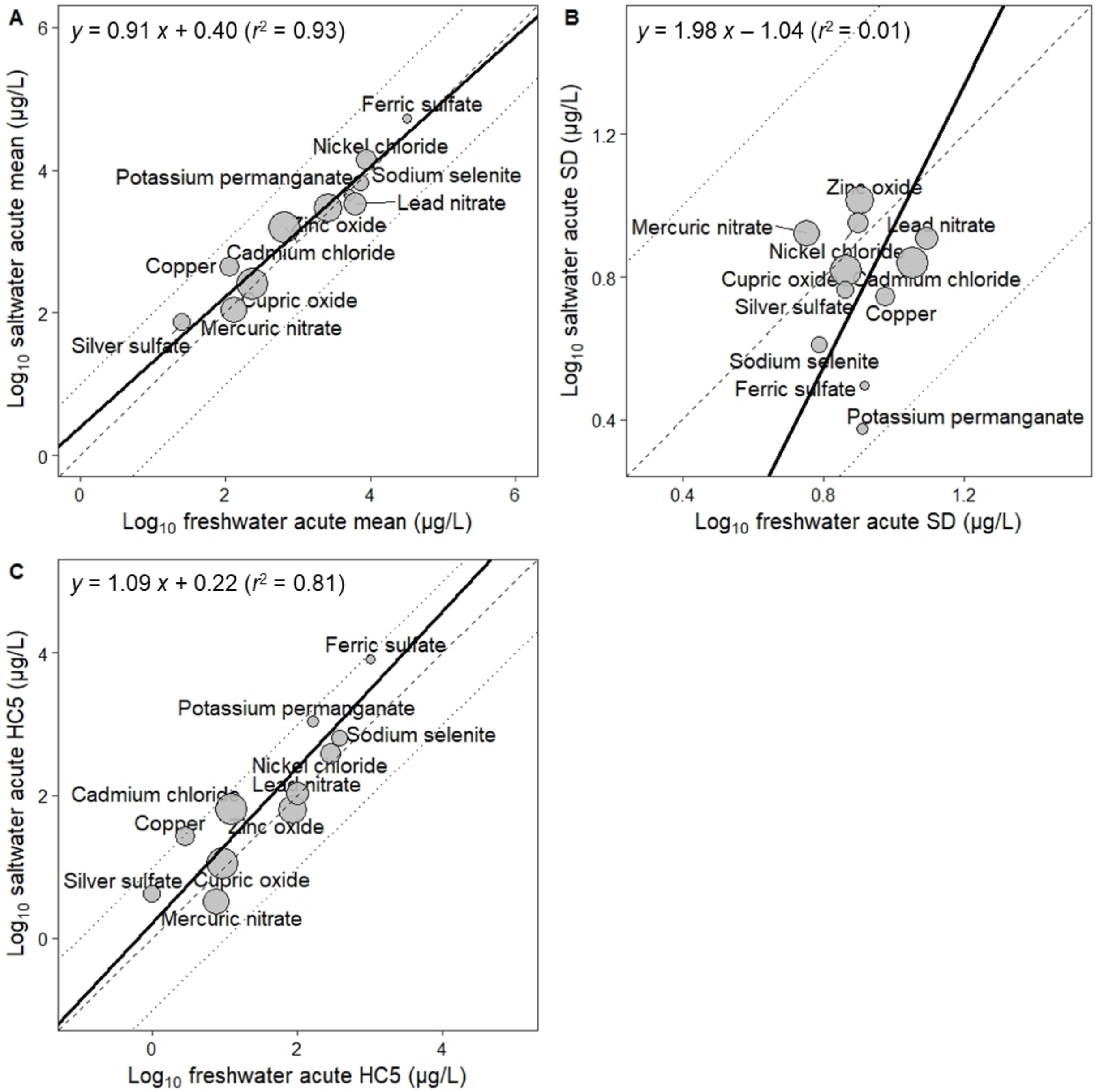

FIGURE S2: Relationship between the (A) means, (B) standard deviations (SDs), and (C) HC5s of freshwater and saltwater SSDs of metals. The SMA regressions of the SSD means, SDs, and HC5s are shown. Details are the same as those in Figure 1. The 95% CI for the slope and intercept were 0.75–1.11 and −0.17 to 0.98 for the means; 0.99–4.00 and −2.40 to 0.33 for the SDs; and 0.79–1.50 and −0.44 to 0.89 for the HC5s.

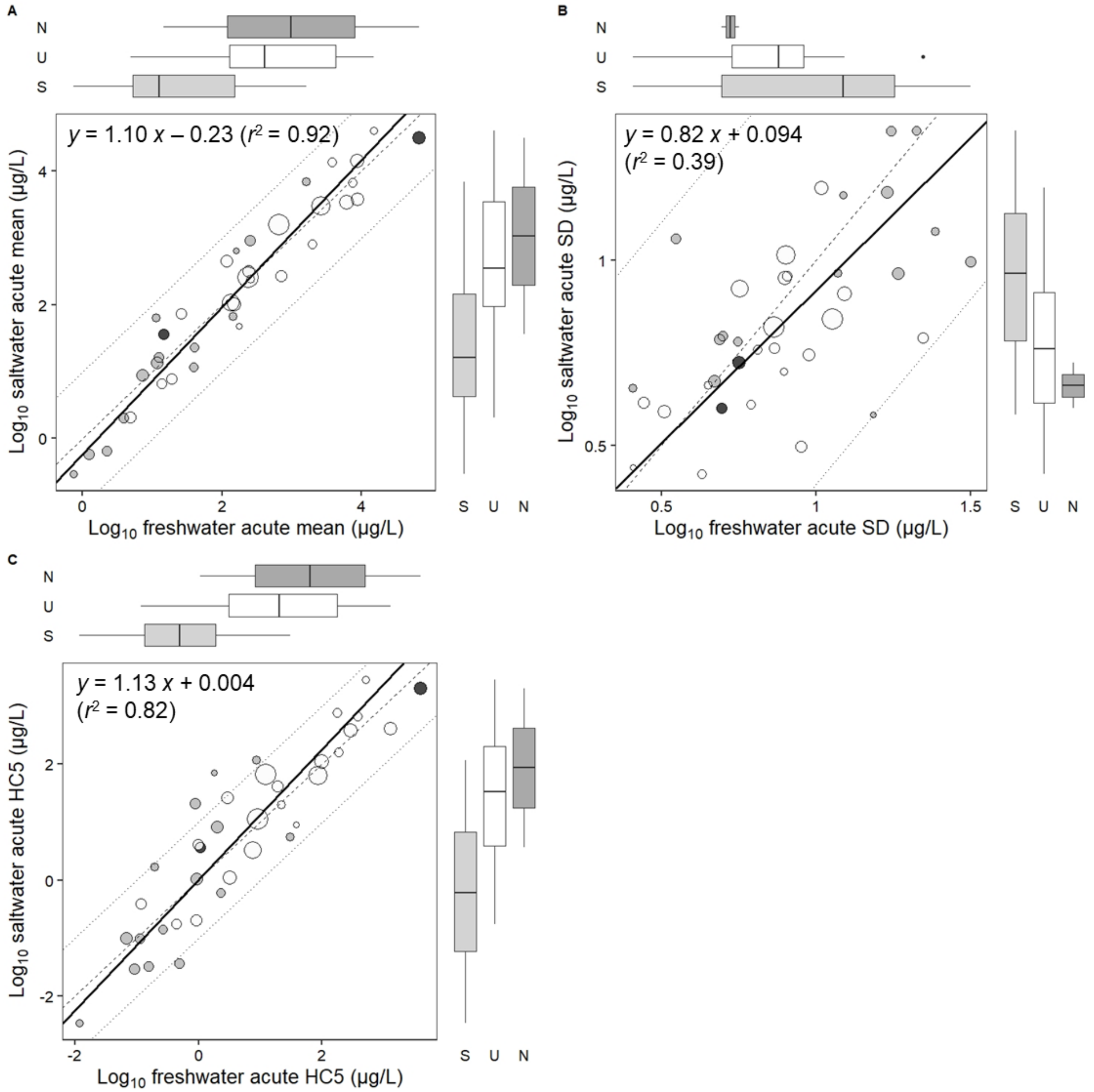

FIGURE S3: Relationship between the (A) means, (B) standard deviations (SDs), and (C) HC5s of freshwater and saltwater SSDs for chemicals tested on at least 10 species. Details are the same as those in Figure 1. The SMA regressions (solid lines) of the SSD means, SDs, and HC5s are shown in each panel. The 95% CIs for the slope and intercept were 1.00–1.21 and −0.49 to 0.04 for the means; 0.63–1.08 and −0.12 to 0.31 for the SDs; and 0.97–1.30 and −0.25 to 0.26 for the HC5s.

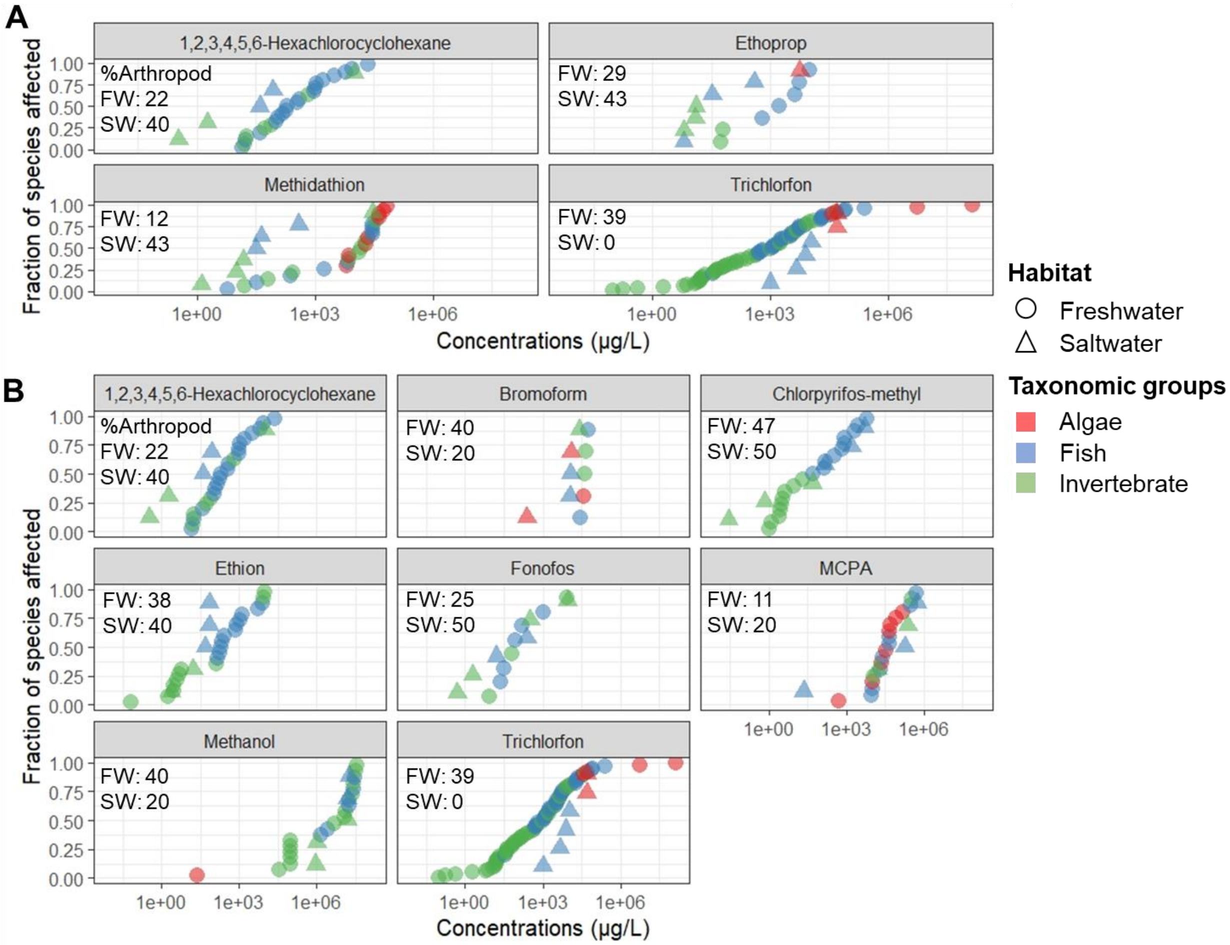

FIGURE S4: Comparison of freshwater and saltwater species sensitivity distributions (SSDs) for chemicals with freshwater:saltwater mean (A) or SD (B) ratios outside the range discussed in Figure 2. Circles and triangles represent freshwater and saltwater effect concentrations, respectively. Dot colors represent the different taxonomic groups of the test species.
